# Pityriasis Lichenoides-Like Mycosis Fungoides: A Case Report

**DOI:** 10.7759/cureus.36665

**Published:** 2023-03-25

**Authors:** Lily Park, Claudia Green, Derrek M Giansiracusa, Penelope Hirt, Carlos Ricotti, Francisco Kerdel

**Affiliations:** 1 Dermatology, Larkin Community Hospital, Miami, USA; 2 Dermatology, Lake Erie College of Osteopathic Medicine, Bradenton, USA; 3 Dermatopathology, Larkin Community Hospital, Miami, USA

**Keywords:** mycosis fungoides, cutaneous t-cell lymphoma, chronic lymphoproliferative diseases, lymphomatoid papulosis, pityriasis lichenoides

## Abstract

A rare subtype of mycosis fungoides (MF) known as pityriasis lichenoides-like mycosis fungoides (PL-like MF) manifests as recurrent crops of erythematous scaly papules with the histological findings of MF. We report a 64-year-old male with recurrent crops of psoriasiform papules with mild scales on his trunk and extremities. Skin biopsy results were consistent with CD8+ cutaneous T-cell lymphoma (CTCL). Our patient had clinical features of pityriasis lichenoides and histological findings consistent with CD8+ MF. A differential diagnosis of PL, lymphomatoid papulosis (LyP), and PL-like MF was considered. Counseling patients with CD8+ cutaneous T-cell lymphoma can be challenging, as there is an aggressive variant named primary cutaneous aggressive epidermotropic CD8+ CTCL. However, with the ability to recognize PL-like MF, a rare indolent type of CD8+ CTCL, physicians can counsel patients appropriately.

## Introduction

Mycosis fungoides (MF) is the most common cutaneous T-cell lymphoma (CTCL) [1]. Diagnosing MF is challenging, as it can present with a variety of clinical features [2]. On histological examination, MF displays an atypical lymphocytic infiltrate with focal epidermotropism [3]. A rare subtype of MF called pityriasis lichenoides-like MF (PL-like MF) manifests as recurrent crops of erythematous scaly papules with the histological findings of MF [1,4]. Ko et al. first described PL-like lesions in three MF patients in 2000 [5].

## Case presentation

**Figure 1:**
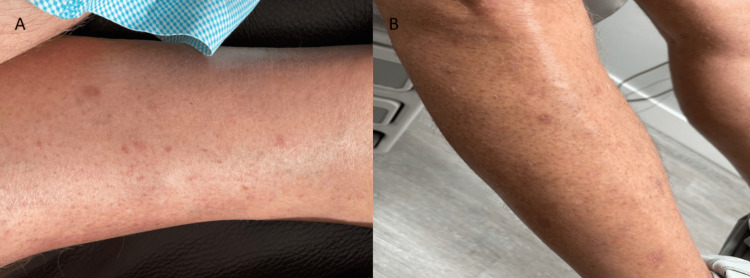
(A and B) Clinical appearance of many scattered erythematous papules on the legs on separate days.

**Figure 2:**
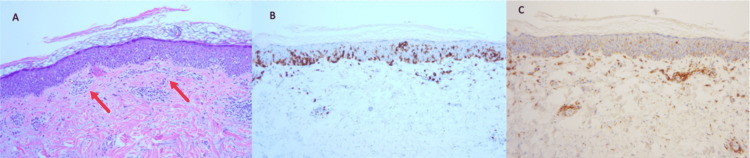
Skin biopsy: histology examination of the skin lesions. (A) H&E staining observed under magnification (10×) reveals atypical lymphocyte exocytosis and perivascular lymphohistiocytic inflammatory infiltrate (arrows) with (B) positive CD8 stain and (C) negative CD4 stain. H&E: hematoxylin and eosin

A 64-year-old Caucasian male with a past medical history of hypothyroidism and mild hypogonadism presented to the dermatology clinic with recurrent erythematous to pigmented, mildly pruritic papules with scant scales on his trunks and extremities for three months (Figure 1). These papules started on the legs and progressed to involve the trunk and upper extremities as more lesions appeared. He had started using intramuscular testosterone, and the rash would appear within half an hour after the injection and would eventually self-resolve in a couple of weeks. A skin biopsy performed by another dermatologist showed exocytosis of atypical lymphocytes and superficial perivascular lymphohistiocytic inflammatory infiltrate along with atypical intraepidermal CD8+ T-cell proliferation consistent with MF. The intraepidermal T-cells were almost exclusively CD8-positive and CD4-negative. The CD3 was positive within the atypical intraepidermal lymphocytes. The CD7 stain demonstrated decreased expression as compared to the CD3 stain within the intraepidermal T-cells. T-cell gene rearrangement studies showed a clonal T-cell population with clonal peaks at 206 bp and 216 bp. His laboratory tests included a complete blood count, comprehensive metabolic panel, thyroid-stimulating hormone, free T4, and antinuclear antibodies, which were all within normal limits. Due to the patient’s high anxiety and the controversial prognosis of CD8+ MF, the patient underwent a PET scan, which was negative. A biopsy was repeated, and the result was consistent with CTCL with a CD4:CD8 ratio of 1:10 (Figure 2). The patient was treated with phototherapy with complete resolution of the lesions with one treatment.

## Discussion

This case had clinical features of pityriasis lichenoides chronica and histological findings of MF. The clinicopathological spectrum of these presentations is wide; therefore, our initial differential diagnosis included pityriasis lichenoides chronica, lymphomatoid papulosis (LyP), and CD8-positive MF, especially PL-like MF. Mycosis fungoides are more prevalent in males and postmenopausal females. Although unconfirmed, testosterone has been thought to be a predictor of this disease [3].

Pityriasis lichenoides (PL) is an idiopathic inflammatory disorder that is most frequently diagnosed in children and young adults and presents as recurrent outbreaks of erythematous papules [4]. Histologically, PL displays an abundance of CD8 cytotoxic/suppressor T-cells typically alongside CD4+ helper T-cells. Frequently, CD3+ T-cells are identified, and in some cases, PL may exhibit CD30 positivity. This makes it challenging to differentiate between PL and lymphomatoid papulosis (LyP), which can have similar clinical and histological findings [4,6]. Our patient presented with CD8+ T-cell proliferation but was CD4-negative. CD30 was unavailable. It is controversial whether or not PL is part of the cutaneous T-cell lymphoproliferative spectrum. Existing cases of PL patients who develop MF raises the question of whether these disorders belong to T-cell lymphoproliferative diseases [4]. If clinical suspicion is raised due to unresponsiveness to treatment for pityriasis lichenoides, serial biopsy with long-term follow-up is recommended with patients off topical therapies for three weeks [5].

LyP has been described to have four clinical variants (A, B, C, and D) based on the distribution of the lymphocytic infiltrate [4]. Differentiating LyP B and the papular variant of mycosis fungoides (PMF) has been an ongoing challenge. PMF is characterized as an early stage of MF that presents with multiple small red-brown papules that are often symmetrically distributed on the trunk and upper and lower extremities [4,7]. In contrast to LyP, the clinical course of PMF is not waxing and waning and morphologically is more uniform and monomorphous [6]. On histology, PMF demonstrates epidermotropism of small to medium atypical lymphocytes with vacuolar interface changes and a band-like lichenoid infiltration of lymphocytes in the papillary dermis [6]. The immunohistochemistry of PMF is identical to MF, demonstrating CD30 negativity. The presence of Pautrier’s microabscesses provides no diagnostic benefit as they may appear in either LyP or PMF. Additionally, CD30 expression is negative in PMF but has variable expression in LyP [8]. Since LyP B is often seen simultaneously with other LyP variants, some authors suggest that the absence of concomitant LyP A-C lesions supports a diagnosis of PMF. Nevertheless, the coexistence of LyP and MF is not unusual [6].

A loss of expression of CD7 has been suggested to be characteristic of MF; therefore, this may provide a useful tool to distinguish between an inflammatory process and neoplasia. However, these results have not been verified as MF has demonstrated CD7 expression in early stages, and inflammatory dermatoses have shown a partial loss of CD7 [4]. Nevertheless, our patient exhibited CD7 negativity, which may suggest a neoplastic process. CD8+ in MF is rare. There is a primary cutaneous aggressive epidermotropic CD8+ CTCL that needs to be distinguished. Since a case of fulminant MF with the CD8+ neoplastic T lymphocytes was published in 1980, there have been other cases of aggressive CD8+ CTCL described in the literature, including 17 CTCL cases with CD8 positivity in 1999 [9,10]. In earlier studies, eight of 17 cases were aggressive with visceral metastases and median survival of 32 months. Histologically, they demonstrated epidermotropism of atypical T-cells with spongiosis and necrosis of keratinocytes and varying degrees of spongiosis and even keratinocytic necrosis, which is unusual in other MF cases. Immunohistochemistry showed CD3 and CD8 positivity with loss of CD2 and CD5 [10]. This aggressive type of CD8+ CTCL is distinguished from other benign types of CD8+ CTCL based on the clinical features of localized or disseminated eruptive papules, patches, or plaques or nodules with hemorrhage, ulceration, and necrosis in the core, natural course of the disease, and physical examination [11]. On the other hand, hypopigmented MF, which tends to appear in young Asians or patients with a darker complexion, also has histological features of atypical intraepidermal predominance of CD8-positive lymphocytes but portends a good prognosis [12]. There have been reports of poikilodermic MF with CD8 positivity, which also has a good prognosis [13]. In addition, Magro et al. observed a benign course of CTCL that evolved from PL, most of which were CD8 lymphocyte-predominant early-stage mycosis fungoides (MF) [14]. They further attributed the indolent nature of these lesions to the cellular cytotoxic response against the aberrant lymphocytes, analogous to the typical course of pityriasis lichenoides [14].

There are few publications on PL-like MF. The first case series was described by Ko et al. in 2000, and a few other authors have published cases since then [4,6,7,15]. Recently, Suh et al. in 2022 described 15 cases of PL-like MF [2]. Among these patients, CD8+ T-cells were more frequently observed (80%). PL-like MF may demonstrate overlapping histological features of both MF and PL. MF features include Pautrier’s microabscess and lichenoid atypical lymphocytes. PL-like features include necrotic keratinocytes, spongiosis, red blood cell (RBC) extravasation, and exocytosis of lymphocytes and neutrophils. However, as in our case, some PL-like MF is diagnosed by clinical manifestations of PL along with pathologic findings of MF rather than histological features of PL. Among the 15 PL-like MF cases, 13 had CD30 negativity. Those with PL-like MF are expected to have a better prognosis than those with other variants of MF. All of the patients were treated with narrowband ultraviolet B (UVB) and experienced complete remission in 73% and recurrence in 17% [2]. Given the good prognosis of PL-like MF, it can be managed similarly to early-stage MF. Oral psoralen and ultraviolet A phototherapy with or without an oral retinoid or narrowband ultraviolet B phototherapy may be used along with topical steroids [2].

## Conclusions

In patients with clinical features of PL and histological findings supporting MF, a differential diagnosis of PLC, LyP, and PL-like MF should be considered. It can be challenging to distinguish between PL, LyP, and variants of papular and PL-like MF without histopathology. In rare cases, PL can evolve into MF. Therefore, patients with a clinical diagnosis of PL are recommended to undergo biopsies when there is suspicion. Counseling patients with CD8+ CTCL can be challenging as a variant of CD8+ CTCL has an aggressive natural course of disease. However, with the ability to recognize a PL-like MF, a rare indolent type of CD8+ CTCL, physicians can counsel patients appropriately.
